# Athletic Intervention After Thoracic Surgery for Lung Cancer: Rationale and Design of the ATHENA Study

**DOI:** 10.1002/hsr2.72512

**Published:** 2026-06-28

**Authors:** Giulia M. Stella, Francesco Bertuccio, Tiziano Gemelli, Matteo Fortunati, Oscar Crisafulli, Mitela Tafa, Amelia Grosso, Annalisa DE Silvestri, Giulio Melloni, Angelo G. Corsico, Giuseppe D'Antona

**Affiliations:** ^1^ Department of Internal Medicine and Medical Therapeutics University of Pavia Medical School Pavia Italy; ^2^ Cardiothoracic and Vascular Department Unit of Respiratory Diseases, IRCCS Policlinico San Matteo Pavia Italy; ^3^ Interdipartimental Centre in Motor and Sport Activities, Sport Medicine Centre University of Pavia Pavia Italy; ^4^ Biostatistics and Clinical Trial Centre, Fondazione IRCCS San Matteo Pavia Italy; ^5^ Department of Public Health, Experimental and Forensic Medicine University of Pavia Medical School Pavia Italy

**Keywords:** aerobic training, early‐stage lung cancer, motor science, structured exercise training, thoracic surgery

## Abstract

**Background:**

The optimal therapeutic approach for early‐stage NSCLC (I–II, and, in some specific cases, IIIa) is lung resective surgery with mediastinal lymphadenectomy. However, it should be remarked that lung resection surgery further negatively affects respiratory muscle function and health‐related quality of life (HRQoL). It is well known that exercise interventions may improve pulmonary function but the optimal approach has not been determined yet.

**Aims:**

The aim of the present study is to investigate the results obtained by administering a specific athletic/physical protocol to a cohort of patients who undergo lung surgery after NSCLC diagnosis.

**Materials and Methods:**

Among patients who underwent a surgical treatment of NSCLC and followed in our Institution, those with the highest scores of performance status, defined after multidisciplinary evaluation by Interdisciplinary Group for Thoracic Neoplasms (GINT) will be addressed to the Interdipartimental Centre in Motor and Sport Activities, Sport Medicine Centre, University of Pavia for a work protocol based on mesocycles of 3times/week for 6 months.

**Results:**

Respiratory function, cardiopulmonary exercise capacity, caloric expenditure expressed as metabolic equivalents (METs), and health‐related quality of life will be assessed at baseline, 3 months, and 6 months in a prospective single‐arm pilot cohort. Caloric consumption will be measured by metabolic equivalents (MET). A quality of life (QoL) questionnaire and a spirometry will be administered to patients at the same intervals.

**Discussion:**

The primary exploratory endpoint will be the change in CPET‐derived VO₂peak from baseline to 6 months; secondary endpoints will include changes in FEV₁, DLCO, MIP/MEP, METs, muscle power, and EORTC QLQ‐C30 scores. For each patient, written informed consent will be obtained before the enrollment.

**Conclusion:**

Our study is, to the best of our knowledge the first perspective clinical trial encompassing athletic programs for patients with surgical treatment of NSCLC. The study is designed to provide preliminary data on feasibility, safety, and functional outcomes of a structured post‐surgical athletic intervention in carefully selected patients with early‐stage NSCLC.

## 1 Introduction

Lung cancer is the predominant cause of cancer cases and deaths and causes significant health challenges. Non‐small cell lung cancer (NSCLC) includes a variety of histologically different tumors. The gold standard therapeutic approach for early‐stage disease is surgery. In patients with an adequate respiratory reserve, lobectomy has been the gold standard; however, recent results of the CALGB and JCOG/WJOC non‐inferiority trials clearly showed the oncological efficacy of a sublobar resection in early‐stage disease. Pneumonectomy is restricted to selected patients in cases of locally advanced, central tumors with invasion of vascular and/or bronchial structures. Surgery for lung cancer patients significantly impacts their quality of life, disability level, and functional profile [as defined by The International Classification of Functioning, Disability and Health (ICF), developed by the World Health Organization (WHO) [1, 2, 3, 4]. Assessing these parameters can aid in better understanding the full impact of surgical intervention, enhancing patient–surgeon communication, improving patient counseling and expectation setting, and monitoring treatment and therapeutic progress. It is reported that multimodal prehabilitation for lung surgery is feasible within a timeframe of 3 weeks. Even though this study was not powered to confirm it, prehabilitation may improve preoperative functional capacity [5]. However, the vast majority of available data regards the effects of Rehabilitation Exercises after thoracic surgery for NSCLC. It has been reported that this approach is effective for managing long‐term persistent postoperative symptoms, such as cough [6]. Post‐surgical rehabilitation improves cardiopulmonary function, encompassing respiratory muscle strength, ventilatory equivalent, tidal volume, stroke volume index, and cardiac index at peak exercise [4]. Some data are still available about physical activity after surgery: for instance, it has been reported that age and surgical procedure affect the increase in post‐operative walking steps [7]. However, data regarding a full training athletic program after surgery are still controversial [8]. Rehabilitation programs are most often reserved to COPD patients or those with lower performance status [9]. Few data is available regarding a full training program based on athletic intervention reserved to highly performant patients, in the absence of comorbidity. We thus aim at validating an athletic training program aiming at improving performance and quality of life.

### 1.1 Impact of Lung Cancer Surgery

Anatomical lung resection remains the standard curative option for patients with early‐stage non‐small cell lung cancer (NSCLC). However, even in individuals with preserved baseline lung function and favorable performance status, surgery often leads to a measurable decline in pulmonary mechanics, cardiopulmonary fitness, muscle strength, and health‐related quality of life (HRQoL). This decline is multifactorial, stemming from pain, reduced ventilatory function, systemic inflammation, muscle catabolism, and prolonged inactivity during recovery [10, 11, 12]. Furthermore, impaired postoperative functional status has been associated with poorer long‐term outcomes, including increased morbidity, delayed return to normal activity, and reduced tolerance to potential adjuvant therapies [13]. Consequently, the postoperative period presents a critical window for targeted interventions aimed at restoring, and ideally enhancing, physical performance.

### 1.2 Role of Respiratory Physiotherapy in Early Recovery

Respiratory physiotherapy has a well‐established role in the prevention of postoperative pulmonary complications, particularly atelectasis and pneumonia. Interventions such as inspiratory muscle training (IMT), positive expiratory pressure (PEP), active cycle of breathing techniques and thoracic expansion exercises have demonstrated benefits in preserving lung volume, improving mucus clearance, and re‐establishing diaphragmatic function [14, 15, 16]. These strategies have been associated with reduced post‐operative complications rates, shorter length of hospital stays, and faster weaning from oxygen therapy [17]. Importantly, early implementation of respiratory physiotherapy enhances patients' readiness to engage in more advanced physical activity, forming the foundation for progressive rehabilitation models.

### 1.3 From Pulmonary Rehabilitation to Progressive Exercise Training

While standard pulmonary rehabilitation programs have historically focused on patients with COPD or limited functional reserve, there is a growing recognition of the need to extend exercise‐based interventions to high‐performance status postoperative cancer patients. The ATHENA study responds to this unmet need by proposing a mesocycle‐based, progressively loaded exercise program, designed to maximize adaptive physiological responses while maintaining safety. ATHENA model does not rely on acute spikes in workload. Instead, it incorporates: (i) structured progression over time, with gradual increases in session complexity and training volume; (ii) supervised aerobic, anaerobic and resistance training, tailored to patient capacity; (iii) integration of motor science principles, including recovery cycles, overload management, and biomechanical optimization, iv) continuous monitoring through VO₂ max, lung function test (FEV₁, DLCO), METs, and standardized HRQoL tools. This structured approach allows for physiological adaptation without exceeding individual safety thresholds an essential consideration in oncologic rehabilitation.

### 1.4 Evidence Supporting Exercise‐Based Rehabilitation: Literature Review

In recent years, structured exercise interventions have been explored to mitigate these challenges and enhance postoperative outcomes.

#### 1.4.1 Preoperative Exercise Training

Preoperative exercise programs have demonstrated significant benefits in reducing postoperative complications and enhancing recovery. A Cochrane systematic review encompassing 10 studies with 636 participants found that preoperative exercise training led to a 55% reduction in the risk of postoperative pulmonary complications compared to standard care. Additionally, these interventions were associated with shorter hospital stays and improved preoperative fitness levels. Further supporting these findings, a meta‐analysis by Cavalheri et al. reported that preoperative exercise training significantly decreased the length of hospital stay by approximately 5 days and reduced postoperative complications. Improvements in exercise capacity, as measured by the 6‐min walk distance (6MWD), were also observed [18, 19].

#### 1.4.2 Postoperative Exercise Training

Postoperative exercise interventions have been shown to enhance functional recovery and quality of life in NSCLC patients. A systematic review and meta‐analysis of randomized controlled trials indicated that postoperative exercise training significantly improved physical and mental health domains of the SF‐36 quality of life questionnaire. However, the effects on exercise capacity and respiratory function were less conclusive, suggesting the need for longer or more intensive training programs. Another meta‐analysis focusing on postoperative recovery highlighted that exercise interventions led to significant improvements in forced expiratory volume in 1 s (FEV₁), quadriceps strength, and alleviation of dyspnea. These findings underscore the role of exercise in enhancing both pulmonary function and overall physical performance during recovery [20, 21, 22].

#### 1.4.3 Combined Pre‐ and Postoperative Exercise Programs

Integrating exercise training both before and after surgery may offer synergistic benefits. A comprehensive review by Mainini et al. emphasized the importance of combining aerobic training with respiratory exercises, such as IMT and airway clearance techniques, to address the multifaceted needs of NSCLC patients. The authors advocate for multidisciplinary rehabilitation programs that are tailored to individual patient profiles, including considerations for comorbidities like chronic obstructive pulmonary disease (COPD) [18, 22].

#### 1.4.4 Implications for Clinical Practice

The accumulating evidence supports the incorporation of structured exercise programs into the perioperative care of NSCLC patients. These interventions not only reduce the risk of postoperative complications but also enhance functional capacity and quality of life. Nevertheless, further high‐quality randomized controlled trials are needed to establish standardized protocols regarding the optimal timing, intensity, and components of exercise programs for this patient population.

## 2 ATHENA Study Design

### 2.1 Study Rationale and Aim

As reported above, there is a strong rationale for rehabilitation after surgery for lung cancer since it impacts on chronic symptoms and lung function. This approach is generally reserved to COPD patients or those with lower performance status [19]. Few data is available regarding a full training program based on athletic intervention reserved to highly performant patients, in absence of comorbidity. We thus aim at validating an athletic training program aiming at improving performances and quality of life. Patients will be selected based on their performance status and on multidisciplinary evaluation by the local Interdisciplinary Thoracic Tumor Board.

### 2.2 Study Design

**Figure 1:**
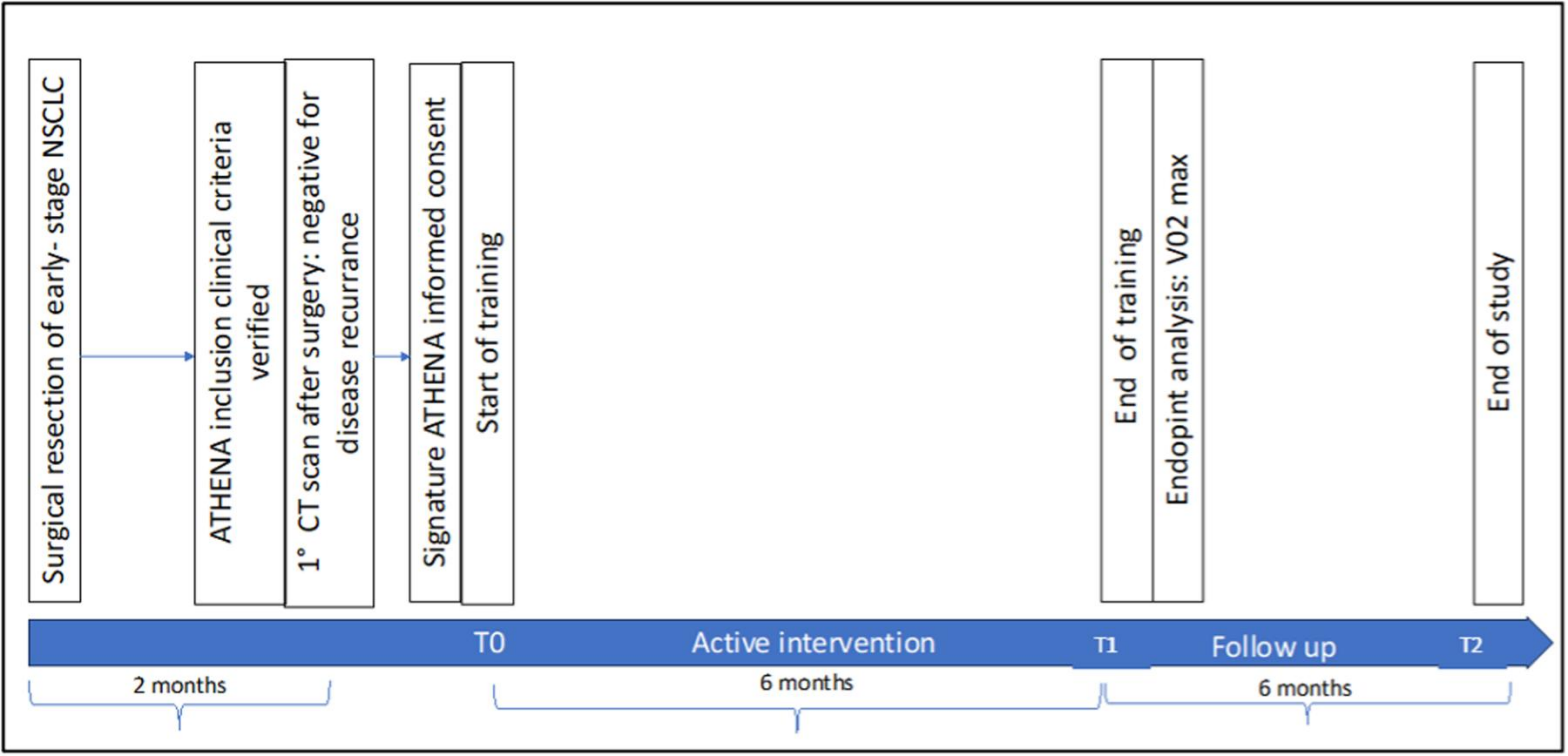
ATHENA study timeline.

This is a single‐center, single‐arm pilot interventional study. The primary purpose of this pilot study is to evaluate the feasibility, safety, and preliminary functional effects of a structured athletic training program administered after curative‐intent lung resection for NSCLC in a highly selected population with preserved performance status. Ten patients will be enrolled and followed for 6 months during the intervention period, with repeated assessments performed at baseline, 3 months, and 6 months (Figure 1).

### 2.3 Study Objectives

The primary exploratory objective of the study is to assess the change in peak oxygen uptake (VO₂peak, mL·kg⁻¹·min⁻¹), measured by cardiopulmonary exercise testing (CPET), from baseline to 6 months in patients undergoing curative‐intent surgery for early‐stage NSCLC and enrolled in the ATHENA training program. VO₂peak/VO₂max reflects the highest oxygen uptake achieved during incremental exercise testing and should not be confused with resting oxygen consumption, whereas 1 MET corresponds to 3.5 mL O₂·kg⁻¹·min⁻¹ at rest. Secondary study objectives are improvement of: (i) the change of pulmonary function (FEV1 and DLCO and VO_2_ max) measured at baseline, at the 3rd and at the 6th month of the study. The latter is defined as the amount of oxygen consumed while sitting at rest and is equal to 3.5 ml O_2_ per kg body weight x min; (ii) The quality of life will be assessed by the EORTC QLQ‐C30 (vers. 3) Questionnaire a 30‐item instrument designed to measure quality of life in all cancer patients [23]; (iii) improvement in caloric consumption measured by metabolic equivalents. Although being a pilot study, demographic exploratory outcomes would be assessed. Results will be analyzed to evaluate if observed differences in outcomes could be related to gender and/or age of the subjects since it is conceivable that the two variables could affect athletic performances and primary and secondary outcomes.

### 2.4 Patients' Identification and Selection

**Figure 2:**
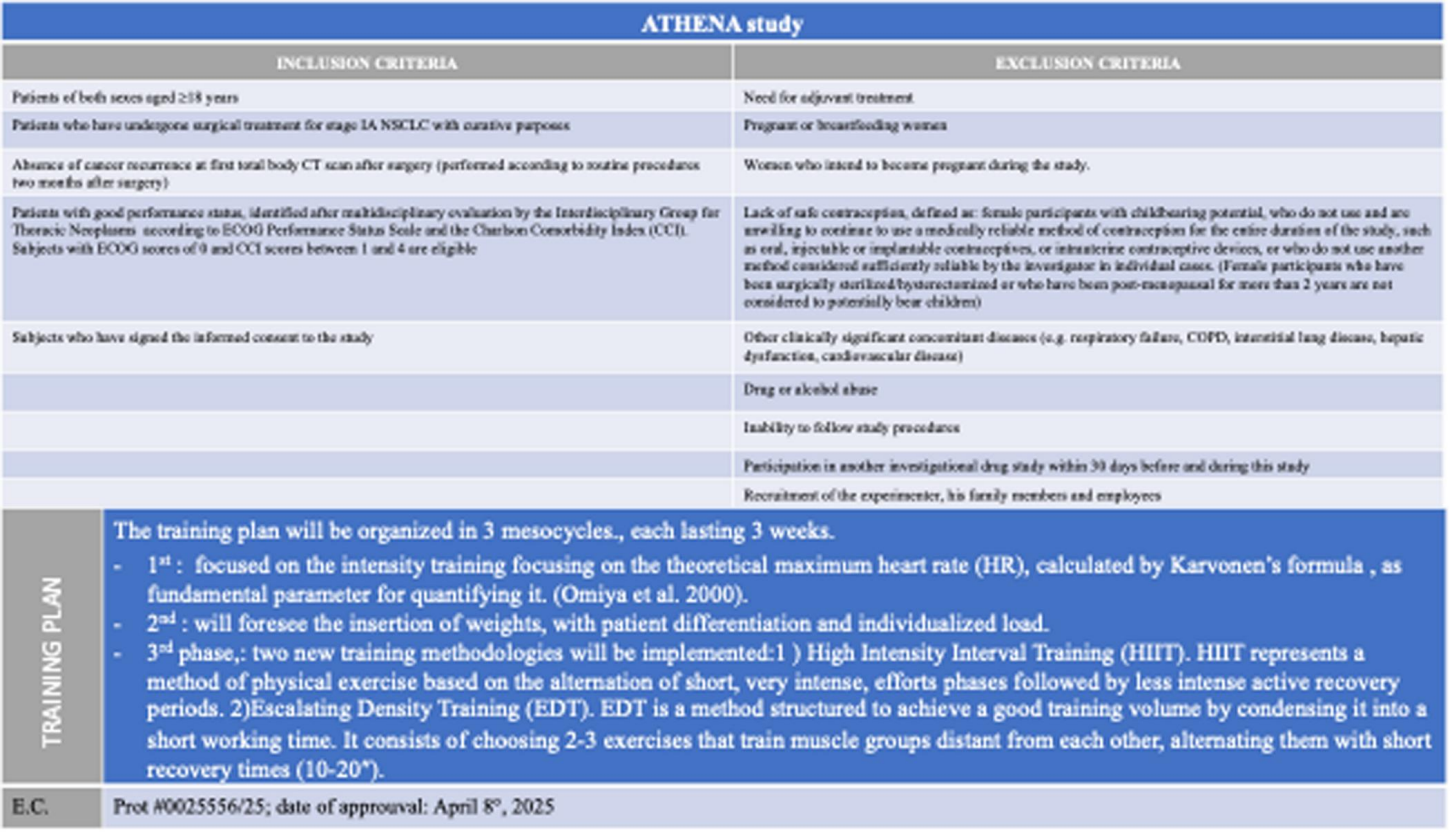
Athena details. Criteria for clinical trial enrollment, training program and ethical approval [24].

Being a pilot study, the study population would be defined by subjects who underwent radical pulmonary surgery for stage IA lung cancer and whose performance status is judged as high after multidisciplinary evaluation by the Interdisciplinary Tumor which is active in our Institution. The population to be studied will be out‐patients subjects followed at the Pneumology Unit after thoracic surgery for lung cancer. To mitigate the study risks, the identification and selection of patients will be defined during the lung cancer board meeting when all lung cancer cases diagnosed and treated at Fondazione IRCCS Policlinico San Matteo and associated spoke centers are discussed: (i) study participants will be recruited at Fondazione IRCCS Policlinico San Matteo, Pneumology Unit among those patients affected by lung cancer and addressed to surgery with a curative setting. After surgery each case will be rediscussed multidisciplinary and patients with higher performance status, defined by ECOG score 0 and CCI score from 1 to 4, will be selected for the study (website https://www.mdcalc.com/calc/3170/eastern-cooperative-oncology-group-ecog-performance-status; https://www.mdcalc.com/calc/3917/charlson-comorbidity-index-cci); (ii) specifically the ECOG Performance Status Scale describes a patient's level of functioning in terms of their ability to care for themselves, daily activity, anphysical ability. An ECOG score of 0 identifies a fully active person, able to perform all pre‐disease services without limitations. The Charlson Comorbidity Index (CCI) is used to classify comorbid conditions which may influence mortality risk. The severity of comorbidity from mild (CCI scores of 1–2) to moderate (CCI scores of 3–4) is used for the selection of patients in the study. Higher scores indicate a more severe condition and, consequently, a worse prognosis. If the patient agrees to participate, the informed consent form will be signed and dated. Written informed consent must be documented before any study‐specific screening procedure. The overall eligibility of the subject to participate in the study will be assessed once all screening values are available (a schematic overview of the clinical timeline and enrollment strategy is provided in Figure 2). Patients meeting all inclusion and none of the exclusion criteria will be enrolled. No payment or compensation will be given to study participants.

#### 2.4.1 Inclusion Criteria

Patients will be considered eligible if they meet all of the following criteria:

– Age ≥ 18 years;

– Histologically confirmed NSCLC treated with curative‐intent lung resection;
– Pathological stage IA disease;
– ECOG Performance Status 0;
– Charlson Comorbidity Index between 1 and 4;
– Clinical stability at approximately 30 days after surgery;
– Ability to undergo CPET and supervised exercise training;
– Ability to understand study procedures and provide written informed consent;
– Absence of contraindications to exercise training according to sports medicine evaluation.

#### 2.4.2 Exclusion Criteria

Patients will be excluded in the presence of any of the following:

– ECOG Performance Status ≥ 1;
– Severe cardiovascular, respiratory, neurological, musculoskeletal, or psychiatric comorbidities limiting safe exercise participation;
– Postoperative complications preventing participation in the training program; ‐Need for adjuvant systemic treatment during the intervention period, if considered incompatible with the exercise protocol;
– Documented disease recurrence before enrollment;
– Inability to adhere to scheduled visits or supervised training sessions;
– Any clinical condition judged by the investigators or sports medicine team to make participation unsafe.

#### 2.4.3 Withdrawal Criteria

Participants may be withdrawn from the study for any of the following reasons:

– Withdrawal of informed consent;

– Occurrence of a serious adverse event or any clinical condition making continued participation unsafe;
– Disease recurrence or initiation of anticancer treatment judged incompatible with protocol continuation;
– Investigator decision based on safety concerns.

All reasons for withdrawal will be recorded.

### 2.5 Statistical Analysis

Categorical variables will be summarized as counts and percentages, whereas continuous variables will be described using mean and standard deviation or median and interquartile range, as appropriate according to their distribution. All statistical analyses will be performed using Stata 18 (StataCorp. 2023. *Stata Statistical Software: Release 18*. College Station, TX: StataCorp LLC.). Given the longitudinal design of the study, outcomes collected at baseline, 3 months, and 6 months will be analyzed using repeated‐measures approaches. Continuous outcomes, including VO₂ peak, pulmonary function parameters, and quality‐of‐life scores, will be primarily analyzed using linear mixed‐effects models, including time as a fixed effect and patient as a random effect, in order to account for within‐subject correlation across repeated measurements. For exploratory analyses, age and sex will be included as covariates in the fixed part of the models. If model assumptions are not met, appropriate generalized linear mixed models or generalized estimating equations will be considered.

Results will be reported as estimated changes over time with 95% confidence intervals. Given the pilot nature of the study and the limited sample size, all efficacy analyses will be considered exploratory and interpreted with caution. A two‐sided alpha level of 0.10 will be used for inferential analyses. Adverse events will be summarized descriptively, and their incidence will be reported with 95% confidence intervals. As a sensitivity analysis, a completer‐only analysis will also be performed.

### 2.6 Detailed Procedure

Before and after the training program, patients will undergo evaluation procedures aimed at investigating exercise capacity, respiratory capacity and muscle power. Both the evaluations and exercise sessions will take place at the CRIAMS Sports Medicine Centre (University of Pavia, Voghera).

The ATHENA intervention was developed through a multidisciplinary collaboration among pulmonologists, thoracic oncologists, sports medicine specialists, motor scientists, thoracic surgeons, and biostatisticians. The protocol was designed on the basis of three elements:

1. Evidence supporting perioperative exercise interventions in NSCLC;
2. The lack of structured post‐surgical programs specifically tailored to patients with high performance status and limited comorbidity;
3. Exercise physiology principles of individualization, progressive overload, recovery, and safety.

The use of 3‐week mesocycles was chosen to allow gradual progression of training load while monitoring tolerance, adherence, and recovery in the early post‐surgical phase.

Each participant will undergo a supervised exercise program three times per week for 24 weeks. The intervention will be delivered at the CRIAMS Sports Medicine Centre under the supervision of trained motor scientists, with medical oversight by sports medicine specialists when appropriate. Each training session will include a warm‐up phase, an aerobic training component prescribed according to the patient's functional evaluation and heart‐rate targets derived from the Karvonen formula and/or CPET results; a resistance and neuromuscular training component tailored to the patient's postoperative status and progressively adjusted across mesocycles and a cool‐down/recovery phase. Training load, exercise intensity, and progression will be individualized according to baseline performance, symptom tolerance, perceived exertion, and interval reassessment at follow‐up visits. Attendance, adherence, temporary interruptions, need for exercise dose reduction, and adverse events will be prospectively recorded.

#### 2.6.1 Evaluation Tests

##### 2.6.1.1 Exercise Capacity Evaluation

The cardiopulmonary exercise test (CPET) is an objective assessment of exercise capacity. The test will take place in the presence of a sports medicine specialist for monitoring cardiac parameters under stress and minimizing the risks inherent the test. The main variable obtainable from the test, i.e. maximal oxygen consumption (VO^2^max), is considered the gold standard of exercise capacity. CPET consists of applying an exercise of gradually increasing intensity until exhaustion or until the appearance of limiting symptoms and/or signs. To apply the increasing load, patients will be asked to pedal on a cycle ergometer (Monark, Cosmed, Italy) and the load will be gradually increased (15 Watts every 2 min) until muscle exhaustion. During the test, the subject will wear a mask to collect respiratory gas breath by breath (Cosmed Quark B2, Cosmed, Italy). In the different phases of the test, the sense of effort will be monitored using a Borg Category Ratio (CR) 0‐10 scale. During the test, cardiac electrical function will be monitored via continuous electrocardiographic recording. At the end of the test, the patient will perform a cool‐down protocol to return to a resting state.

##### 2.6.1.2 Respiratory Capacity Evaluation

Forced spirometry will be carried out to evaluate respiratory capacity. The measurement will be as follows: patient will stand in an upright position, a clip will be applied on the nose and a mouthpiece, equipped with a disposable antibacterial filter, will be placed in the mouth. After a few spontaneous breaths, the patient will be asked to perform a forced inhalation followed by a forced exhalation and a new forced inhalation. The patient will be asked to repeat the procedure three times after a pause of a few seconds. Spirometry will be performed using a Cosmed spirometer. To evaluate the maximum inspiratory pressure (MIP) and the maximum expiratory pressure (MEP) a PoinX pressure monitor (Cosmed) will be used. The procedure will involve wearing the nose clipper, positioning the mouthpiece equipped with a disposable antibacterial filter and performing three maximal inhalations and three maximal exhalations for a few seconds at constant pressure.

##### 2.6.1.3 Muscle Power Evaluation

To evaluate muscle power, a bench press set of three repetitions will be performed on a Smith Machine (Multipower, Technogym, Cesena, Italy). Repetitions will begin with a rapid but controlled eccentric movement, immediately followed by a straight explosive vertical push. All the repetitions will be performed continuously, without intra‐set rest. A linear position transducer (LPT) (MuscleLabTM, Model 4000e, Ergotest Technology, Norway) with a sampling frequency of 200 Hz will be used to record vertical displacement (cm) and execution time (milliseconds); subsequently, on the basis of these data, mean and peak velocity (m/s) and power (W) output will be calculated.

#### 2.6.2 Training Plan

The training plan will be organized in mesocycles, according to the following scheme. Of note, each mesocycle will last 3 weeks. The theoretical maximum heart rate (HR), fundamental parameter for quantifying the intensity of training, will be calculated by Karvonen's formula [25].

#### 2.6.3 Procedures at Each Visit

During the course of the study, visits and assessments will be performed as defined in Section 9.1. For all measurements, the actual date and time of assessment will be recorded in the Source Document and in the eCRFs. All the exams will be performed at the coordinator center for all visits and follow‐up. Each patient will enter the study 30 days after surgery. The first screening visit will encompass spirometry, DLCO analysis and VO_2_ max calculation. Patients will also undergo an electrocardiogram (ECG) as per clinical practice, standard routine blood exam and clinical visit. Then the patient will enter the trial and subsequent controls, similar to the first one will be done at 3rd and 6th month, at the end of the program. Radiological surveillance will be performed according to routine oncologic follow‐up and institutional practice and will not be considered a study‐specific outcome measure of the ATHENA intervention. After the end of the active interventional study, patients will continue the standard follow‐up procedures according to national and international guidelines.

### 2.7 Study Risks and Adverse Events

The above‐described approach will assure the identification of patients presenting at lower risks for the program. Risks related to the program itself encompass muscular damages and bone fractures. To minimize them, the enrolled subjects will be supervised by dedicated motor scientists during each step. Adverse events may include: (i) musculoskeletal injuries: ‐sprains and strains: injuries to muscles, tendons, and ligaments.‐ stress fractures: microscopic fractures that can occur due to repetitive loading‐ tendinitis: inflammation of the tendons—joint pain: inflammation and injuries to joints, such as runner's knee or swimmer's shoulder; (ii) cardiovascular problems:‐ myocardial Infarction: especially in sedentary individuals who start an intense exercise program—cardiac arrhythmias: irregular heartbeats that can be triggered by physical exercise—sudden cardiac death: a rare but possible event, often associated with undiagnosed heart conditions. Moreover, in some individuals, physical exercise can trigger respiratory problems, including exercise‐induced asthma or bronchospasm that occurs during or after exercise, hyperventilation that can lead to feelings of dizziness and tingling.

## 3 Expected Results

The ATHENA study‐protocol aims to address a critical unmet need and introduces an innovative paradigm in the postoperative care of NSCLC patients: a mesocycle‐based, progressive physical training program delivered under expert supervision and tailored to individuals with high performance status. By leveraging principles of exercise physiology, motor science, and clinical oncology, the protocol aims not only to restore functional capacity but to actively enhance it, with anticipated improvements in VO₂ max, pulmonary function, and patient‐reported quality of life. Unlike traditional rehabilitation or structured exercise training, ATHENA proposes a graduated, integrative model, drawing from sports medicine, oncology, and pulmonary care to deliver personalized and evidence‐based post‐surgical intervention. The combination of respiratory physiotherapy and structured athletic training offers a multidimensional strategy to optimize pulmonary function, cardiovascular fitness, and quality of life. If results will be achieved and validated, our protocol could set the stage for a broader transformation in thoracic oncology survival where exercise prescription is individualized, progressive, and function‐driven, marking a shift from recovery to performance optimization.

## 4 Conclusions

Cancer is a complex condition characterized by a close interconnection between genetic drivers and the immune surrounding context. A large amount of data indicates that in some critical issue cancer is intimately linked to aging and cellular senescence which can also affect tumor progression by acting on genomic instability and chronic inflammation [for a review see [25, 26, 27]. In this scenario, exercise can be seen as a powerful intervention able to target not only lung function and quality of life of lung cancer patients but also on ageing and biologic basis of disease progression. In this perspective, the ATHENA trial is expected to provide preliminary evidence on the feasibility, safety, and functional impact of a structured post‐surgical athletic intervention in selected patients with early‐stage NSCLC, thereby informing the design of larger prospective studies.

## Author Contributions

**Giulia M. Stella:** conceptualization, investigation, writing – review and editing, validation, methodology, project administration, supervision, writing – original draft, funding acquisition, visualization, formal analysis, data curation. **Francesco Bertuccio:** conceptualization, investigation, writing – original draft, methodology, validation. **Tiziano Gemelli:** conceptualization, methodology, validation, visualization, writing – review and editing, supervision. **Matteo Fortunati:** writing – review and editing, visualization, validation, data curation. **Oscar Crisafulli:** validation, visualization, writing – review and editing, data curation. **Mitela Tafa:** writing – review and editing, visualization, validation, data curation. **Amelia Grosso:** methodology, validation, visualization, writing – review and editing, formal analysis, project administration. **Annalisa DE Silvestri:** methodology, software, formal analysis, project administration, writing – review and editing, validation, visualization. **Giulio Melloni:** validation, visualization, writing – review and editing, data curation. **Angelo G. Corsico:** supervision, data curation, writing – review and editing, validation, visualization. **Giuseppe D'Antona:** supervision, data curation, investigation, writing – review and editing, visualization, methodology, validation.

## Disclosure

The lead author Francesco Bertuccio affirms that this manuscript is an honest, accurate, and transparent account of the study being reported; that no important aspects of the study have been omitted; and that any discrepancies from the study as planned (and, if relevant, registered) have been explained.

## Ethics Statement

The study will be conducted in accordance with the Declaration of Helsinki and was approved by the Local Ethics Committee. This study involves human participants and was approved. The study was submitted as part of a main project that was approved by the local Ethical Commission, and each enrolled patient gave written informed consent before enrollment (Comitato di Bioetica, Fondazione IRCCS Policlinico San Matteo, approval numbers: protocol #20090002344; procedure # 20090019080; date of approval: 3 June 2009). The research was conducted according to the principles of the World Medical Association Declaration of Helsinki. Participants gave informed consent to participate in the study before taking part.

## Consent

Consent obtained directly from patient(s).

## Conflicts of Interest

The authors declare no conflicts of interest.

## Data Availability

The data that support the findings of this study are available on request from the corresponding author. The data are not publicly available due to privacy or ethical restrictions.
