# Correction to “Athletic Intervention After Thoracic Surgery for Lung Cancer: Rationale and Design of the ATHENA Study”

**DOI:** 10.1002/hsr2.73115

**Published:** 2026-08-30

G. M. Stella, F. Bertuccio, T. Gemelli, et al., “ Athletic Intervention After Thoracic Surgery for Lung Cancer: Rationale and Design of the ATHENA Study,” *Health Science Reports* 9 (2026): e72512, https://doi.org/10.1002/hsr2.72512.

The name of the eighth author was misspelled and has been updated from Annalisa di Silvestri to Annalisa DE Silvestri.

The online version of this article has been corrected accordingly

We apologize for this error.

